# President’s Address

**Published:** 1906-09-15

**Authors:** J. J. Green


### PRESIDENT'S ADDRESS.

BY J. J. GREEN, D. D. S.

Read to the Michigan Dental Association, July 9, 1906.

Members of the Michigan Dental Association—We are assembled for the Semi-Centennial Meeting of this Association. The object of this organization was two-fold; professional advancement and fraternity. Of the first we have acchieved a measure of success far greater than our charter member's highest ambitions. With professional advancement the second object has kept an even pace, and to-day we are more determined than ever to carry out the plans and ambitions of that first meeting a few loyal and liberal men.

If the efforts of your officers should be supported by each and every member of the Association, our membership can be increased and with benefits to all. If there is an ethical dentist present who is not now a member, he ought not to leave the meeting until he has registered his application with the Secretary. It is not enough for you to be a passive attendant at these meetings, you need to be an active member, and should try to induce every ethical dentist with whom you come in contact to give the association the strength of his assistance.

Specializing in our profession is increasing every year and is placing it on a higher and broader basis each year. As an association we must create in our passive brethren more interests for the profession, which aims at the best good to our patients. The specialized practice of Orthodontia, Porcelain and Dental Prophylaxis have done much to develope men of a high order of skill, who by their works should stimulate the general practitioner who has a wider field for advancement.

Dental Society meetings are really post graduate courses for interested dentists. The papers, discussions and clinics enthuse us with the possibilities before us; and create a deeper interest as well as a desire to contribute our share to the general progress.

Every effort has been put forth by the Trustees and Board of Directors to make this meeting one of, if not the best in the history of this Association. We are anxious that the surviving charter members should see how much we wish to carry out their purposes and ambitions. Upon this fiftieth anniversary it was decided that the best tribute of respect we could show would be the holding of this meeting in Detroit, the birth-place of the organization. Hence the postponement until another year of the Saginaw meeting.

The Detroit Dental Society has again amply proven, it usefulness in furnishing this very fine place for our meetings and assures us that the Detroit dentists consider it no imposition, but an honor to have us with them again this year.

We have only two surviving charter members. Dr. A. T. Metcalf and Dr. Isaac Douglass both of whom we are pleased to have with us today. We are unfortunate in the loss of the record book of the Association for the first twenty-five years, I would suggest that a committee be appointed to compile for us as correct a record for these years as memory can substitute, as it can better be done now than later on. It should then be put in print so that it will be permanently preserved.

Under the state law now in force our Governor has full power to appoint whom he wishes, as members of the Board of Dental Examiners. The Board of Directors at the regular annual business meeting in March, suggested five names of reputable and ethical practitioners, whom they believed capable and willing, properly to fill the office which will become vacant in November. They believe this to be for the best interests of the profession as it would tend to divorce the appointments from politics, and we hope that the Governor may see fit to appoint some one of them.

You, no doubt are aware that there is under consideration a movement for a new dental law, and it has been suggested that as representatives of the profession we should ask for improvements in the laws which control the practice of dentistry in our state. It is common knowledge that Michigan's dental law has a low standard as compared with the best dental laws of other states. This is not speaking very well for Michigan with two dental colleges of high standing. I would recommend the amendment of our by-laws, so that the action heretofore taken by the Directors in nominating candidates for the State Board will be in harmony with the constitution of this organization. I would also recommend that we appoint two of our members to act with a member appointed by the Board of Examiners, and two already appointed by the South-west Michigan Society, and one from each of the Dental Colleges, who shall meet and draft a new bill for amending our law regulating the practice of dentistry and for advocating it in the coming session of the Legislature. I would suggest that the actual expenses of travel and attendance on such committee work be paid out of the Association treasury.

It is the desire of the present Directors that the public professional work of the profession in the state shall be systematically done. To this end a committee has been at work all year making plans to organize the dental societies of the state, so that a better grade of work shall be undertaken, and that opportunities may be made for the profession to gather in small and larger conventions at convenient places throughout the entire state. To this end an address will be made before this meeting by Dr. A. D. Black, who has brought the entire state of Illinois into a satisfactory organization, and increased the membership of that society to nearly three times its former strength. It has met with such general approval that other states are planning to adopt the scheme and we trust you may be interested to hear Dr. Black and be enthused to join in such plans as may be adopted to carry this work throughout our state.

You will notice by the programme that the Trustees have appointed assistants so that there will be no difficulty in knowing just when and where each part of the programme is being carried out.

I specially request that section 6 of the Constitution be complied with, in so far as, it provides that "No one shall be permitted to address the chair before giving his full name and address, and it has been announced by the chair." This will greatly assist the secretary in keeping a correct record of the events of the meeting.

I wish to cordially commend the trustees who by their wisdom and energy have provided so excellent a program, and I also wish to commend the secretary for the artistic souvenier which we have in the printed program. I have no doubt but that we shall always keep it as a pleasant reminder of this meeting,.

